# Identify miRNA-mRNA regulation pairs to explore potential pathogenesis of lung adenocarcinoma

**DOI:** 10.18632/aging.204341

**Published:** 2022-10-19

**Authors:** Xingchen Fan, Xuan Zou, Cheng Liu, Shuang Peng, Shiyu Zhang, Xin Zhou, Jun Zhu, Wei Zhu

**Affiliations:** 1 Department of Geriatrics, The First People’s Hospital of Lianyungang, The Affiliated Lianyungang Hospital of Xuzhou Medical University, The Affiliated Hospital of Kangda College of Nanjing Medical University, Lianyungang 222002, P.R. China; 2 First Clinical College of Nanjing Medical University, Nanjing 210029, P.R. China; 3 Department of Gastroenterology, First Affiliated Hospital of Nanjing Medical University, Nanjing 210029, P.R. China; 4 Department of Oncology, First Affiliated Hospital of Nanjing Medical University, Nanjing 210029, P.R. China; 5 Department of Radiation Oncology, Nanjing Medical University Affiliated Cancer Hospital, Jiangsu Cancer Hospital, Jiangsu Institute of Cancer Research, Xuanwu, Nanjing 210009, P.R. China

**Keywords:** miRNA, miRNA-mRNA regulation pairs, lung adenocarcinoma

## Abstract

Purpose: MicroRNA (miRNA) function via base-pairing with complementary sequences within mRNA molecules. This study aims to identify critical miRNA-mRNA regulation pairs contributing to lung adenocarcinoma (LUAD) pathogenesis.

Patients and methods: MiRNA and mRNA microarray and RNA-sequencing datasets were downloaded from gene expression omnibus (GEO) and the cancer genome atlas (TCGA) databases. Differential miRNAs (DE-miRNAs) and mRNAs (DE-mRNAs) were screened by the GEO2R tool and R packages. DAVID, DIANA, and Hiplot tools were used to perform gene enrichment analysis. The pairs of miRNA-mRNA were screened from the experimentally validated miRNA-target interactions databases (miRTarBase and TarBase). External validation was carried out in 30 pairs of LUAD tissues by quantitative reverse transcription and polymerase chain reaction (qRT-PCR). The diagnostic value of the miRNA-mRNA regulation pairs was evaluated by receiver operating characteristic curve (ROC) and decision curve analysis (DCA). Biological function assay was were also performed to confirm the function of miRNA-mRNA axis in LUAD progression. The study also performed the clinical, survival and tumor-associated phenotypic analysis of miRNA-mRNA pairs.

Results: A total of 7 miRNA and 13 mRNA expression datasets from GEO were analyzed, and 11 DE-miRNAs (5 down-regulated and 6 up-regulated in LUAD tissues) and 128 DE-mRNAs (30 up-regulated and 98 down-regulated in LUAD tissues) were identified. The pairs of miR-1-3p(down) and CENPF(up) and miR-126-5p(down) and UGT8(up) were verified in the external validation cohort (30 LUAD vs. 30 NC) using qRT-PCR. Areas under the ROC curve of the two miRNA-mRNA regulation pairs panel were 0.973 in TCGA-LUAD and 0.771 in the external validation. The DCA also showed that the miRNA-mRNA regulation pairs had an excellent diagnostic performance distinguishing LUAD from normal controls. The expression of the regulation pairs is different in different ages, TNM stages, and gender. The overexpression of miR-1-3p and miR-126-5p significantly inhibited the proliferation and migration of LUAD cells. Correlation analysis showed that CENPF correlated with prognosis and tumor immunity.

Conclusions: The research identified potential miRNA-mRNA regulation pairs, providing a new idea for exploring the genesis and development of LUAD.

## INTRODUCTION

Lung cancer has the highest incidence and mortality of all cancers [1, 2]. Lung adenocarcinoma (LUAD) is a major component of lung cancer, accounting for 40% of lung cancer [3]. Although the oncology treatment of advanced lung cancer has made significant progress in recent years, the 5-year survival rate remains poor. Therefore, further studies on the underlying mechanism of tumor initiation and development are necessary.

MicroRNA (miRNA) is a class of short non-coding RNA molecules ranging from 19 to 25 nucleotides [3–5]. MiRNAs work by base-pairing with complementary sequences within the mRNA molecule [6, 7]. More and more researches are focusing on the miRNA-mRNA regulation pairs, trying to explore the mechanism of the pairs in the occurrence and development of the disease [8–10].

The research performed an extensive analysis of miRNA-mRNA regulatory pairs in LUAD to provide a new strategy for the underlying mechanism of LUAD.

## MATERIALS AND METHODS

### miRNA and mRNA expression profiles

**Figure 1:**
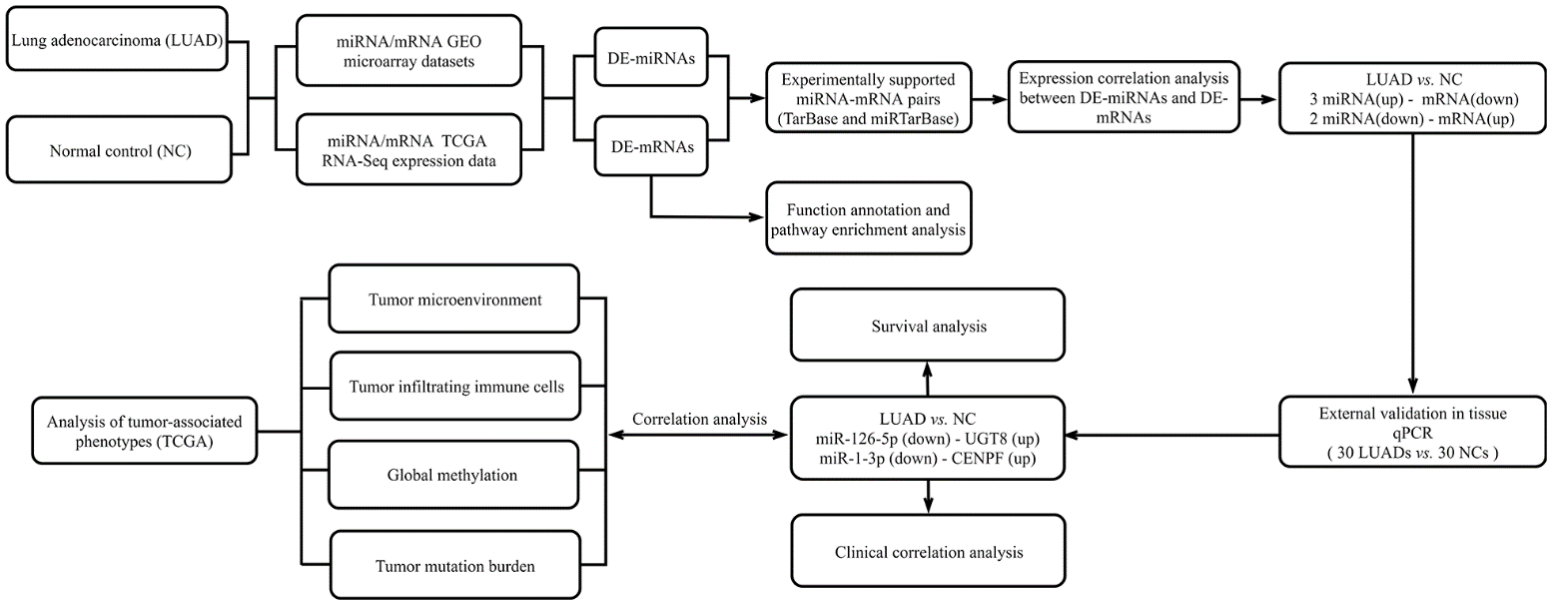
Flow chart for identifying the miRNA-mRNA regulatory networks and the comprehensive analysis of regulatory pairs role in lung adenocarcinoma (LUAD).

We downloaded the miRNA and mRNA expression profile from the TCGA database and the Gene Expression Omnibus (GEO) database. We use the GEO database web analytics tool GEO2R and “limma” and the “edgeR” R bag to filter DE-mRNAs and DE-miRNAs. The overview of the workflow steps is shown in Figure 1.

### Analysis of miRNA-mRNA regulation pairs

TarBase and miRTarBase databases were used to screen miRNA-mRNA regulatory pairs. TarBase and miRTarBase are experimentally supported miRNA target index reference databases [11, 12]. Then, we further analyzed the correlation between miRNA and mRNA in TCGA-LUAD. We used DAVID, Diana-miRPath and Hiplot for functional and pathway analysis [13].

### Sample collection

Formalin-fixed paraffin-embedded (FFPE) of LUAD and corresponding normal tissues were obtained from the First Affiliated Hospital of Nanjing Medical University. This study was conducted in accordance with the Helsinki Declaration and approved by the Institutional Review Committee of the First Affiliated Hospital of Nanjing Medical University (ID: 2016-SRFA-148). All specimens were collected with informed written consent of patients. The clinical characteristics of the 30 patients are listed in Table 1.

**Table 1:** Clinicopathological and molecular features of LUAD patients.

**Variables**	**Number of cases**	**Rate (%)**
	**(n=30)**	
**Age (years)**		
≤60	14	46.7
>60	16	53.3
**Gender**		
Female	21	70
Male	9	30
**Tumor size (cm)**		
≤3	21	70
> 3	9	30
**TNM stage**		
I	21	70
II-IV	9	30
**Lymph node metastasis**		
No	21	70
Yes	9	30
**Bronchial invasion**		
No	24	80
Yes	6	20

### Quantitative reverse transcription PCR (qRT-PCR) assay

External validation of qRT-PCR validation was performed using PrimeScript RT reagent Kit (Takara) and SYBR Premix Ex Taq II (Takara). PCR primer sequences are shown in Supplementary Table 1. We used the 2^-ΔΔCt^ to calculate miRNA and mRNA expression levels (ΔCt = Ct_miRNA/mRNA_− Ct_normalizer_; Ct: the threshold cycle) [14].

### Cell culture and cell transfection

Lung adenocarcinoma cell lines A549 was obtained from the American Type Culture Collection (ATCC). The cells were seeded into 24-well plates. The miR-126-5p mimics, miR-1-3p mimics, Negative control mimics were purchased from RiboBio. When cell fusion reached 60%, cells were transfected with 20mM Opti-MEM transfection medium (Invitrogen) and Lipofectamine 2000(Invitrogen).

### Cell proliferation and scratch wound healing assays

Cell Counting Kit-8 (CCK-8, Dojindo, Kumamoto, Japan) assay was used to assess cell proliferation. At indicated time points (24h, 48h, 72h, 96h), the cells were incubated in 10% CCK8 solution in culture medium at 37° C. The absorbance at 450nm was measured with a microplate reader. To examine the migratory ability of cells *in vitro*, the scratch wound healing assay was performed. When the cells were cultured to 80%-90% in 6-well plates, after the medium was discarded, the cells were scratched with 100 μL tip. The cells were placed in serum-free DMEM medium and observed at 0 and 24h.

### Analysis of tumor-related phenotypes

We downloaded the data of single sample gene set enrichment analysis (ssGSEA) from UCSC Xena [15, 16]. The infiltrating immune cell types data were downloaded from the TCGA website [17]. ESTIMATE software was used to evaluate the stromal and immune levels of TCGA-LUAD specimens [18]. The data of TMB and methylation in TCGA-LUAD samples were obtained from the UCSC Xena platform (https://xena.ucsc.edu/) [19].

### Statistical analysis

We used the IBM SPSS Statistics v.26 software, GraphPad Prism software and R language v3.6.3 (https://cran.r-project.org/) to analyze the data.

### Data availability statement

The data that support the findings of this study are available from the corresponding author upon reasonable request.

## RESULTS

### Screening of differentially expressed miRNA and mRNA

**Figure 2:**
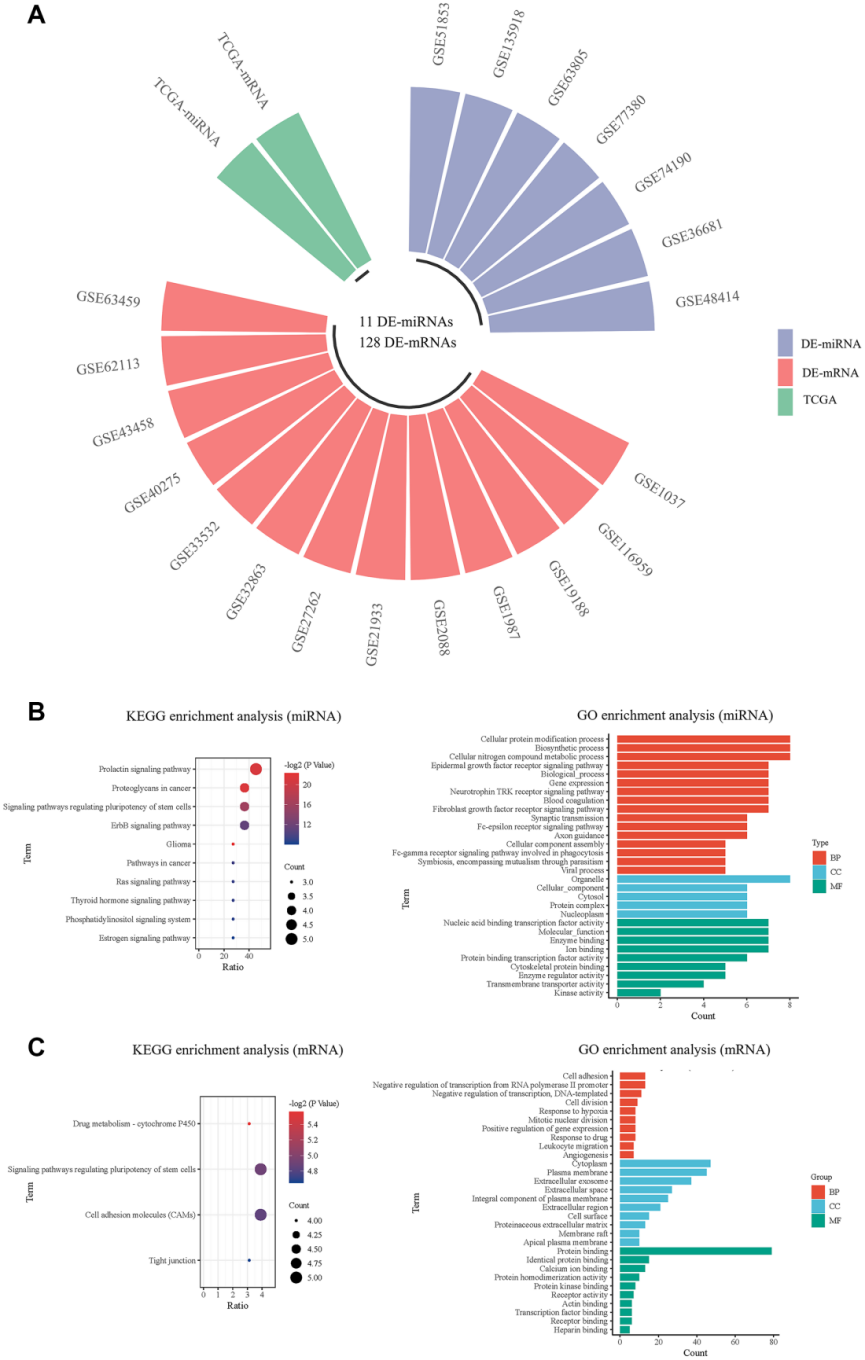
**Screening and pathway analysis of DE-miRNAs and DE-mRNA.** (**A**) The circular bar chart showing the datasets from different sources for screening differentially expressed miRNAs and mRNAs; (**B**) The KEGG and GO enrichment analysis of DE-miRNAs; (**C**) The KEGG and GO enrichment analysis of DE-mRNAs.

A total of 7 miRNA and 13 mRNA expression datasets were downloaded from GEO database, and the information of 20 GEO datasets is shown in Table 2. As shown in Figure 2A, the GEO2R tool was used to analyze each dataset, and the DE-miRNAs and DE-mRNAs in each dataset were screened out. Then, the intersection was taken in the GEO database. A rank-sum test was performed to screen out the DE-miRNAs and DE-mRNAs in the TCGA database. A total of 11 miRNAs and 128 mRNAs were selected with differences in both databases as the final DE-miRNAs and DE-mRNAs (Table 3). We utilized DIANA-miRPath to predict the possible functions of the 11 DE-miRNAs (Figure 2B). KEGG pathway enrichment analysis revealed that the DE-mRNAs enriched in the drug metabolism, etc. (Figure 2C). The GO terms were enriched in the cell adhesion, cellular protein modification process, cytoplasm, organelle, etc.

**Table 2:** Information pertaining to the selected GEO datasets for LUAD.

	**Experiment type**	**Source name**	**GEO accession**	**Platform**	**Group**	
					**Tumor**	**Control**
**microRNA expression**	Array	Tissue	GSE51853	GPL7341	76	5
			GSE135918	GPL18058	5	5
			GSE63805	GPL18410	32	30
			GSE77380	GPL16770	3	12
			GSE74190	GPL19622	36	44
			GSE36681	GPL8179	103	103
			GSE48414	GPL16770	154	20
**mRNA expression**	Array	Tissue	GSE1037	GPL962	12	19
			GSE116959	GPL17077	57	11
			GSE19188	GPL570	45	65
			GSE1987	GPL91	7	7
			GSE2088	GPL962	9	30
			GSE21933	GPL6254	11	21
			GSE27262	GPL570	25	25
			GSE32863	GPL6884	58	58
			GSE33532	GPL570	40	20
			GSE40275	GPL15974	8	43
			GSE43458	GPL6244	40	30
			GSE62113	GPL14951	7	9
			GSE63459	GPL6883	33	32

**Table 3:** The list of DE-miRNAs and DE-mRNAs (up-regulated or down-regulated in LUAD).

**DE-miRNA (down)**	**DE-mRNA(up)**	**DE-miRNA(up)**	**DE-mRNA(down)**
hsa-miR-139-5p	ANLN	hsa-miR-182-5p	ABCA8
hsa-miR-30a-3p	AURKA	hsa-miR-183-5p	ABLIM3
hsa-miR-486-5p	BAIAP2L1	hsa-miR-196a-5p	ACADL
hsa-miR-1-3p	BUB1	hsa-miR-96-5p	ADAMTSL3
hsa-miR-126-5p	CCNA2	hsa-miR-135b-5p	ADARB1
	CCNB1	hsa-miR-9-5p	ADH1B
	CDCA7		AFAP1L1
	CEACAM1		AGER
	CEACAM5		AHNAK
	CENPF		AQP4
	CTHRC1		BMP2
	CXCL13		BMPR2
	EZH2		BTNL9
	FHL2		CADM1
	HIST1H2BD		CALCRL
	HMGA1		CAT
	KIF11		CAV1
	METTL7B		CBX7
	PDIA4		CD34
	PGM2L1		CD36
	PLOD2		CD93
	PTTG1		CDH5
	S100P		CLDN18
	SPP1		CLEC1A
	STK39		CLIC5
	SULF1		CPB2
	TK1		CYBRD1
	TPX2		CYYR1
	UGT8		DACH1
	XPR1		DPT
			EDNRB
			EFEMP1
			EMCN
			FABP4
			FAM107A
			FBLN1
			FBLN5
			FCN3
			FEZ1
			FGD5
			FHL1
			FHL5
			FMO2
			FZD4
			GAS6
			GATA2
			GKN2
			GNG11
			GPC3
			GPM6A
			GPM6B
			GRASP
			GRK5
			GSTM5
			ID3
			IGSF10
			KLF4
			LDLR
			LMO2
			LMO7
			MAL
			MAOB
			METTL7A
			MFAP4
			MME
			MYH10
			MYH11
			MYL9
			PDE8B
			PDZD2
			PLEKHH2
			PODXL
			PRELP
			PTPRB
			PTPRM
			RAMP2
			RHOJ
			SASH1
			SCGB1A1
			SDPR
			SFTPC
			SH2D3C
			SLC39A8
			SLIT2
			SNRK
			SOSTDC1
			SPARCL1
			STARD13
			STX11
			STXBP6
			TBX2
			TEK
			TGFBR3
			THBD
			TIMP3
			TMEM47
			VWF
			WIF1

### Screening of miRNA-RNA regulatory pairs

**Figure 3:**
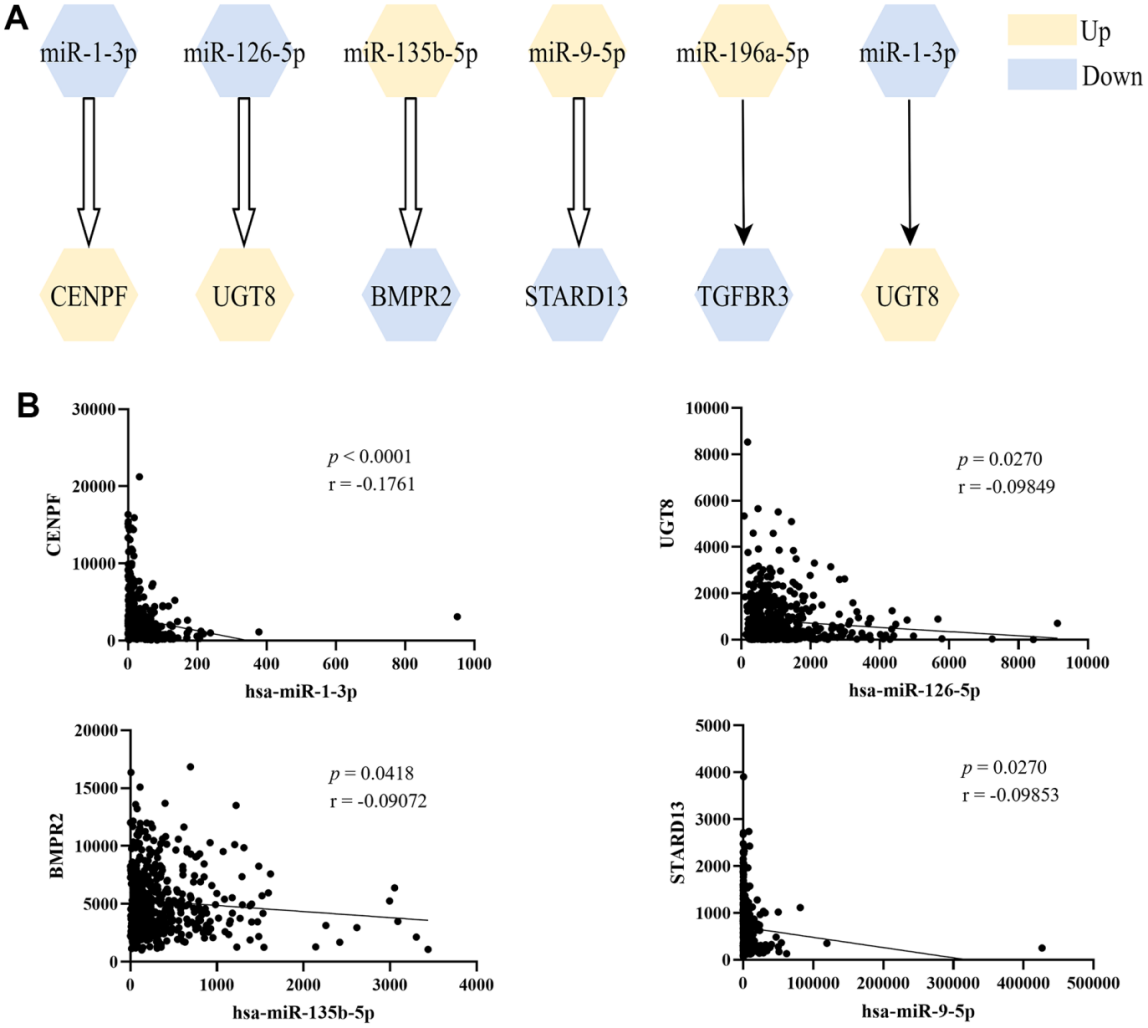
**The screened miRNA-mRNA regulation pairs.** (**A**) Six miRNA-mRNA regulatory pairs were screened from miRTarBase and TarBase databases, and the first four were verified by correlation analysis; (**B**) Pearson’s correlation analysis of miRNA-mRNA regulation pairs in TCGA-LUAD.

As shown in Figure 3A, six miRNA-mRNA regulation pairs (miR-1-3p/CENPF, miR-126-5p/UGT8, miR-135b-5p/BMPR2, miR-9-5p/STARD13, miR-196a-5p/TGFBR3, miR-1-3p/UGT8) were identified. The 6 pairs of miRNA-mRNA were experimentally verified, and the 4 miRNA-mRNA pairs in TCGA-LUAD showed significant negative correlation (Figure 3B).

### Verification of miRNAs and mRNAs expression in LUAD tissues

**Figure 4:**
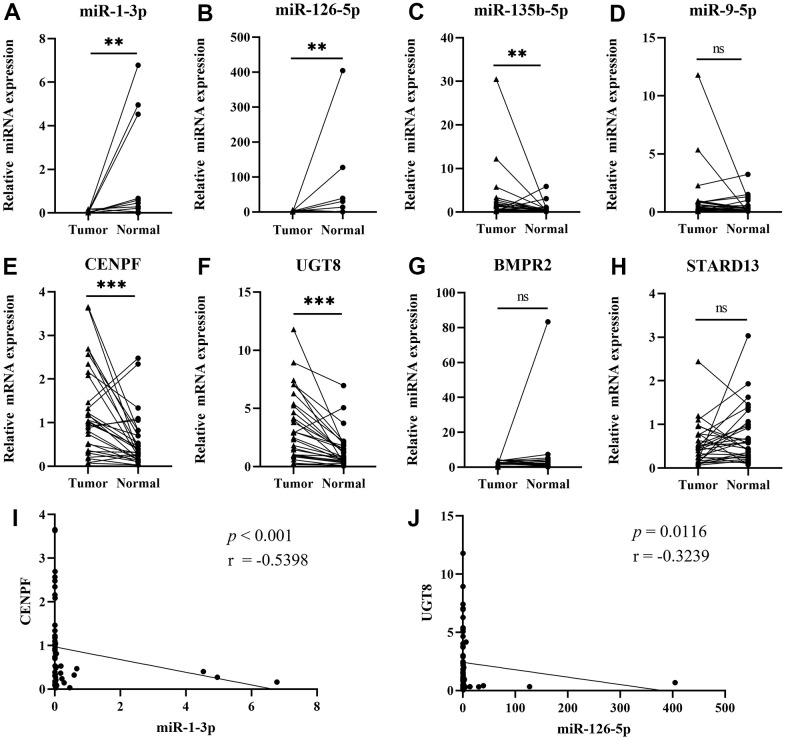
**Validating the expression of the four networks by qRT-PCR.** (Data are presented as mean±SEM; *p < 0.05, **p < 0.01, ***p < 0.001). (**A**) miR-1-3p; (**B**) miR-126-5p; (**C**) miR-135b-5p; (**D**) miR-9-5p; (**E**) CENPF; (**F**) UGT8; (**G**) BMPR2; (**H**) STARD13; (**I**) Pearson’s correlation analysis of miR-1-3p and CENPF; (**J**) Pearson’s correlation analysis of miR-126-5p and UGT8.

We used qRT-PCR to verify 4 miRNA-mRNA pairs in 30 matched tissues. In Figure 4, the expression of the miR-1-3p (*P*=0.0037) and miR-126-5p (*P*=0.0032) were down-regulated in tumor tissues, while miR-135b-5p (*P*=0.0037), CENPF (*P*<0.001) and UGT8 (*P*<0.001) were up-regulated. There was no significant difference in the expression of miR-9-5p (*P*=0.0841), BMPR2 (*P*=0.4522), and STARD13 (*P*=0.1241). Spearman correlation analysis showed that miR-1-3p was significantly correlated with CENPF expression (*P*<0.001, r=-0.5398), and miR-126-5p was significantly correlated with UGT8 (*P*=0.0116, r=-0.3239). IHC images in the HPA database evidenced higher expression of CENPF and UGT8 in LUAD tissue than in normal control and the results are shown in Supplementary Figure 1.

### Analysis of the diagnostic efficacy

**Figure 5:**
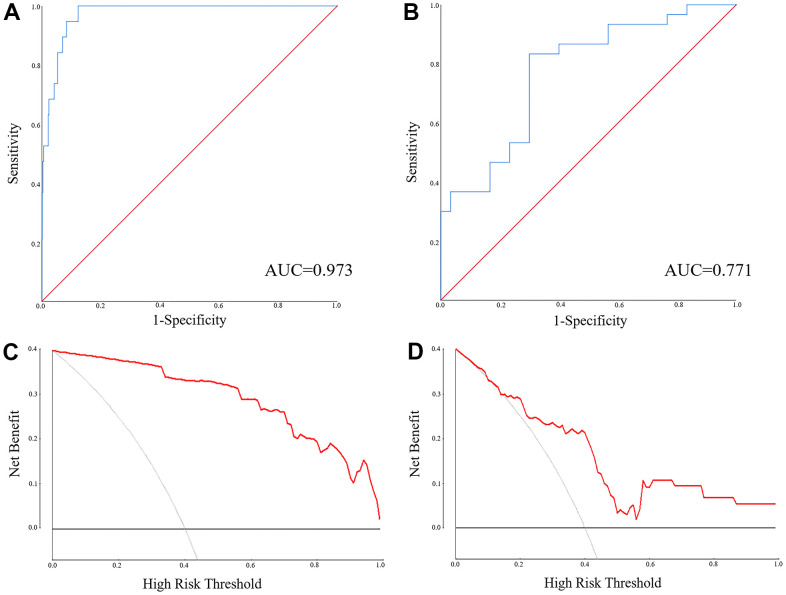
**The ROC and DCA of the panel of miR-1-3p, miR-126-5p, CENPF and UGT8 for discriminating LUAD patients from NCs.** (**A**) The ROC of the TCGA-LUAD (AUC = 0.973, 95% CI: 0.955-0.991, *p*<0.0001); (**B**) The ROC of the external validation (AUC = 0.771, 95% CI: 0.652-0.890, *p*<0.0001); (**C**) The DCA of the external validation; (**D**) The DCA of the TCGA-LUAD.

MiR-1-3p, PTPRM, miR-126-5p and UGT8 were combined as a panel using the logistic regression analysis, and the equation to predict LUAD probability was: Logit(P) = 0.813 + 0.028*miR-126-5p – 0.262*UGT8 + 1.727*miR-1-3p – 0.526*CENPF. The AUC of the panel was 0.973 (95% CI: 0.955-0.991, *p*<0.0001) in TCGA-LUAD and 0.771 (95% CI: 0.652-0.890, *p*<0.0001) in the external validation (Figure 5A, 5B). The DCA results showed that regulation pairs had good diagnostic performance (Figure 5C, 5D).

### Correlation analysis of LUAD clinical-pathological features and survival analysis

**Figure 6:**
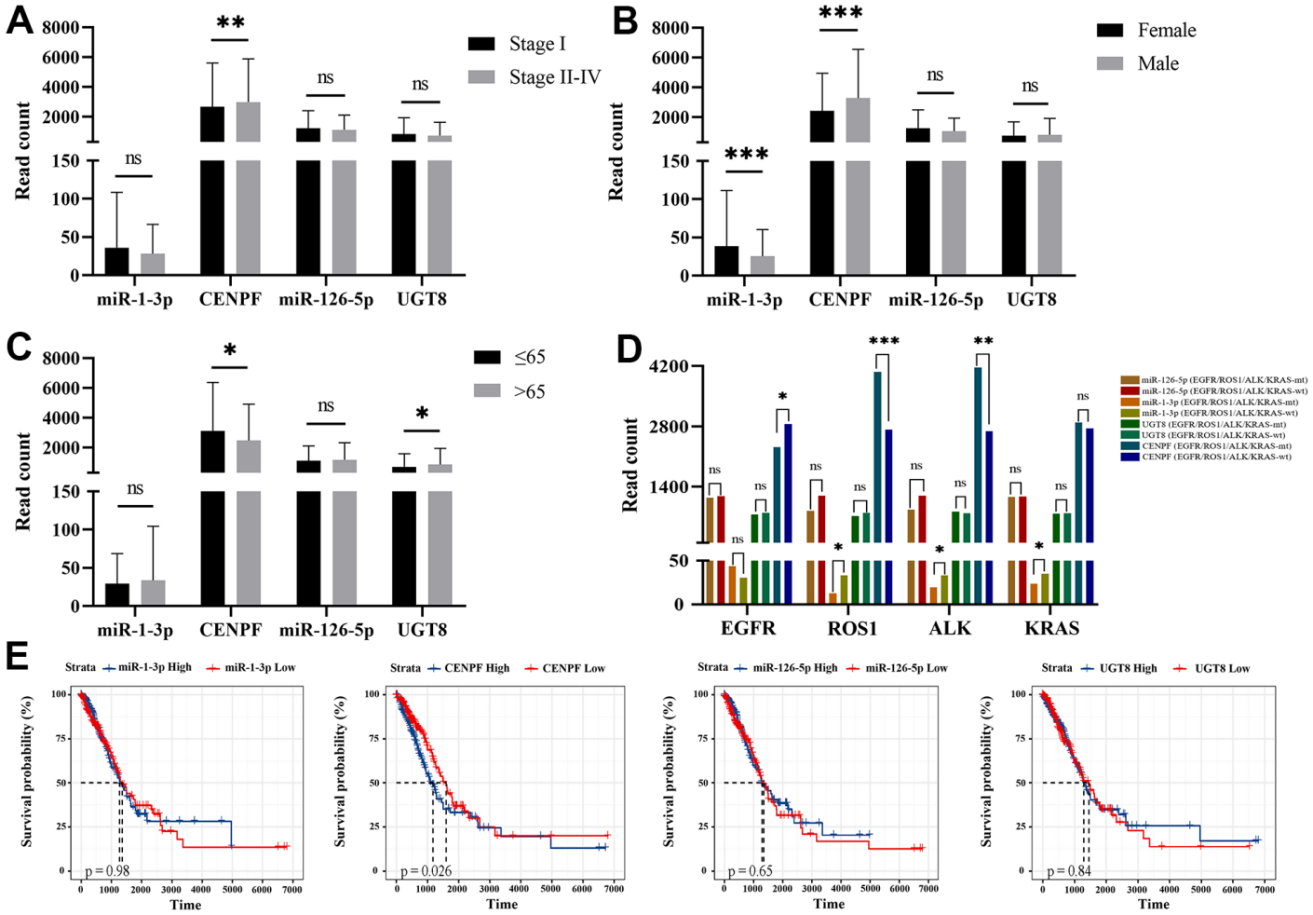
**Correlation analysis of LUAD clinical-pathological features and survival analysis with the expression of miRNA and mRNA expression levels.** (Data are presented as mean±SEM; *p < 0.05; **p < 0.01; ***p < 0.001). (**A**) Stage I vs. Stage II-IV; (**B**) Female vs. Male; (**C**) Age ≤ 65 vs. Age > 65; (**D**) mutation vs. wild-type of KRAS, ROS1, ALK, EGFR; (**E**) The survival analysis of miR-1-3p, CENPF, miR-126-5p and UGT8 in TCGA-LUAD.

According to the analysis of FIGO stages, the expression of CENPF (*P*=0.008) is lower in early-stage (I) than late-stage (II+III+IV) (Figure 6A). The expression of miR-1-3p (*P*=0.001) in female patients is higher, while CENPF (*P*=0.001) was lower in female patients (Figure 6B). CENPF was higher in the age≤65 group while UGT8 was lower in the age≤65 group (Figure 6C). We analyzed the association of the regulation pairs and gene mutations in KRAS, ROS1, ALK, and EGFR. The level of miR-1-3p was higher in KRAS(*P*=0.039), ROS1(*P*=0.013), ALK(*P*=0.02) wild-type LUAD tissues than KRAS-mutated LUAD tissues (*P*=0.039, *P*=0.013, and *P*=0.02, respectively). The expression of CENPF was higher in ROS1-mutated, ALK-mutated LUAD tissues and EGFR wild-type LUAD tissues (*P*<0.001, *P*=0.008, and *P=*0.041, respectively) shown in Figure 6D. K-M survival analysis showed that CENPF (*P*=0.026) correlated with prognosis, and the higher the CENPF expression level, the worse the prognosis (Figure 6E).

### Validation of cell experiments

**Figure 7:**
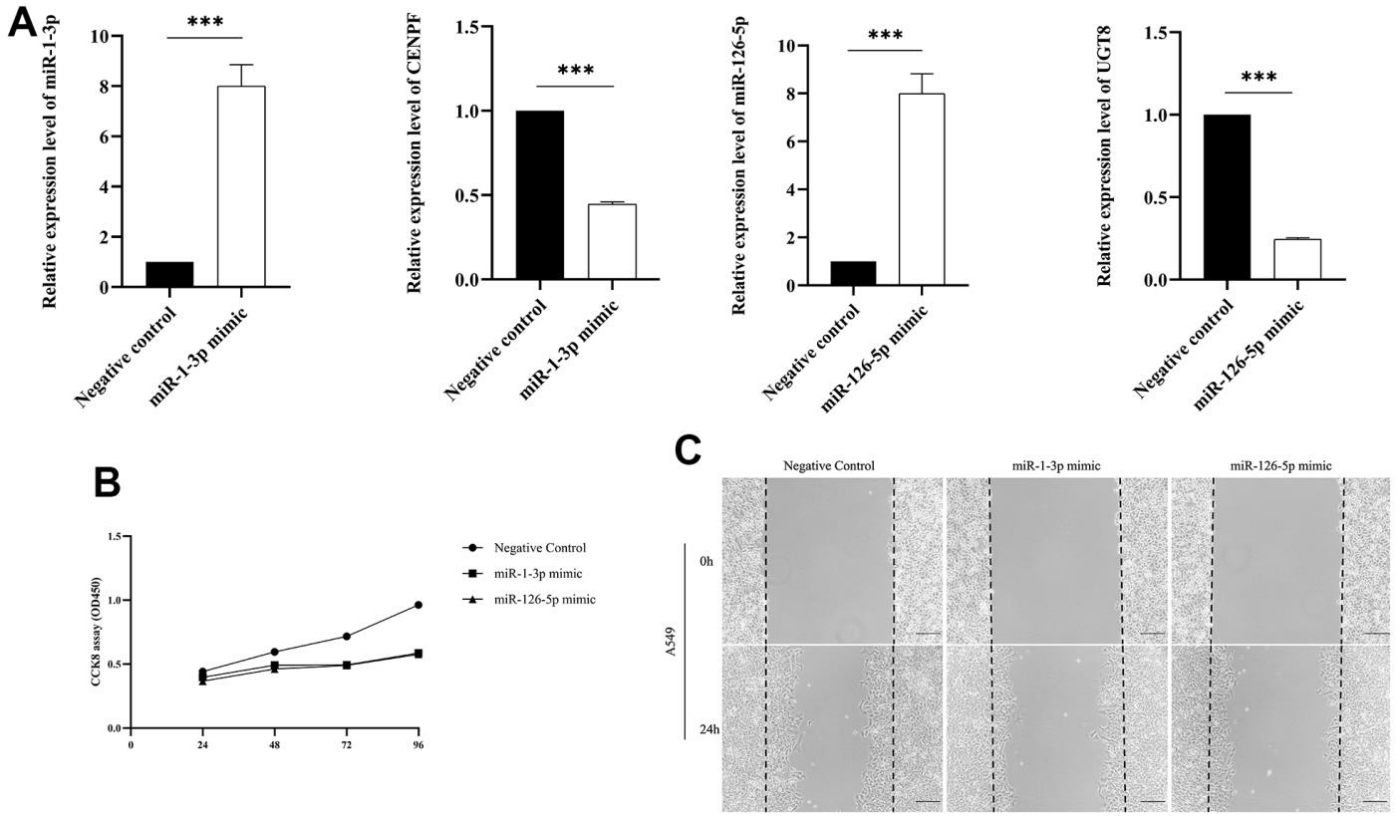
**The validation of biological function assays.** (Data are presented as mean±SD; *p < 0.05; **p < 0.01; ***p < 0.001). (**A**) Comparison of miRNA and mRNA expression levels between transfected miR-1-3p mimic and miR-126-5p mimic and negative control; (**B**) CCK-8 assay was performed to assess cell proliferation; (**C**) Wound-healing assay was conducted to explore LUAD cell migration.

We transfected miR-1-3p mimic and miR-126-5p mimic into A549 cells to established miR-1-3p and miR-126-5p overexpressed cells to investigate the potential function in regulating LUAD cell proliferation. The expression level of miR-1-3p and miR-126-5p in A549 cells upregulated significantly after transfecting miR-1-3p and miR-126-5p mimic (Figure 7A). We found that the mRNA levels of CENPF and UGT8 were declined after transfecting miR-1-3p mimics and miR-126-5p mimics respectively (Figure 7A). Then CCK8 assay was performed to testify the effects of miR-1-3p and miR-126-5p on cell proliferation. MiR-1-3p and miR-126-5p overexpressed significantly inhibited cell proliferation of A549 cells after transfecting 48h, 72h and 96h (Figure 7B). To gain further insight into the role of miR-1-3p and miR-126-5p in LUAD cell migration was performed in A549 cells transfected with miR-1-3p and miR-126-5p mimics or Negative control. The overexpression of miR-1-3p and miR-126-5p significantly inhibited LUAD cell migration (Figure 7C).

### Analysis of tumor-related phenotypes

**Figure 8:**
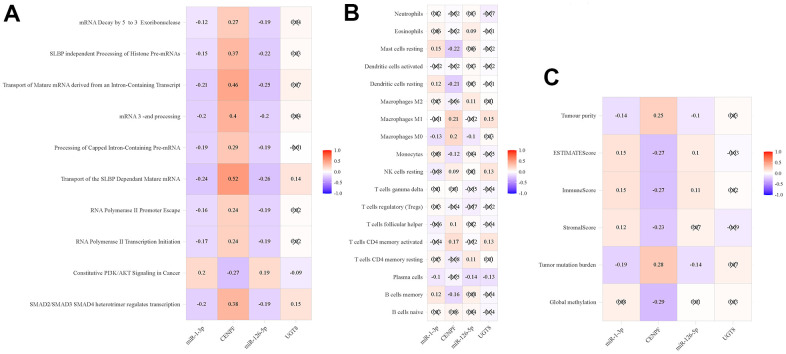
**Pearson’s correlation analysis of immune-related phenotypes and regulatory pairs in TCGA-LUAD.** (**A**) ssGSEA; (**B**) Immune cells; (**C**) Global methylation, tumor mutation burden and tumor microenvironment factors.

The miRNA-mRNA regulation pairs correlated with mRNA synthesis pathways, such as transport of the SLBP dependent mature mRNA (Figure 8A). Therefore, we further analyzed the correlation between miRNA-mRNA regulation pairs and immune cells, and explored its role in tumor immunity. There are 19 different types of immune cells between tumor tissue and normal tissue, as shown in Supplementary Table 2. MiR-1-3p and CENPF correlated with macrophages m0, mast cells resting, and dendritic cells resting, etc. while miR-126-5p and UGT8 were related to plasma cells (Figure 8B). As shown in Figure 8C, miR-1-3p and CENPF have some correlation with TMB and tumor microenvironment, but not with DNA methylation.

## DISCUSSION

In the past few years, many studies have suggested that changes in the expression levels of miRNA and mRNA are closely related to cancers [20–22]. The research aims to construct potential miRNA-mRNA regulatory pairs in LUAD. Firstly, we selected qualified datasets from the GEO database and determined 7 miRNA and 13 mRNA datasets. Expression profiles in the GEO datasets and TCGA database were analyzed using GEO2R, “R-limma” and “R-edgeR” tools to screen for DE-miRNA and DE-mRNA. 11 DE-miRNAs (6 upregulated and 5 down-regulated miRNAs) and 270 DE-mRNAs (30 upregulated and 98 down-regulated mRNAs) showed consistent differential expression in the TCGA database and 7 miRNA and 13 mRNA datasets of the GEO database. The verified pairs were screened from miRTarBase and TarBase database, and Pearson correlation analysis was performed on TCGA-LUAD to screen out 4 miRNA-mRNA regulatory pairs. We further verified the expression levels of 4 pairs in 30 pairs of FFPE lung tissues by qRT-PCR. Finally, the pairs of miR-1-3p-CENPF and miR-126-5p-UGT8 were verified.

In this study, miR-1-3p was low expressed in LUAD tissues and was different in different genders. Studies have shown that miR-1-3p is significantly down-regulated in human LUAD tissues and acts as a suppressor in LUAD cells [23, 24]. Overexpression of CENPF has been reported to have poor prognosis and metastasis of breast cancer, lung adenocarcinoma and prostate cancer [15, 25, 26]. In the research, the expression of CENPF was higher in LUAD tissues and higher in late-stage (II+III+IV) compared with early-stage (I). Patients with higher CENPF expression had worse prognosis. Our study found that miR-126-5p was lower while UGT8 was higher in LUAD tissue. miR-126-5p plays an important role in regulating apoptosis, invasion, migration and EMT of NSCLC cells [27]. A previous study reported that UGT8 is a molecular marker associated with lung cancer metastasis [28].

The correlation analysis between ssGSEA and miRNA-mRNA regulation pairs indicated that these two miRNA-mRNA regulation pairs were related to the synthesis and processing of RNA and mRNA. MiR-1-3p targeting CENPF affects the tumor microenvironment through infiltrating interactions with tumor-associated inflammation, macrophages, mast cells, dendritic cells, and B cells. Therefore, CENPF has an important relationship with tumor immunity.

KRAS, ROS1, ALK, and EGFR are the main biomarkers affecting clinical practice of lung cancer [29–31]. KRAS mutations are present in 30% of lung adenocarcinomas and lead to activation of the Ras-Raf-MEK-ERK signaling pathway, making it an attractive target for small molecule inhibition in KRAS mutant NSCLC [32]. ROS-1 chromosomal rearrangement defines novel genomic driver in 1-2.5% of NSCLC patients [33]. The product of EML4-ALK is detected in 3–6% of unselected NSCLC [34, 35]. In this study, we discovered that miR-1-3p is down-regulated in KRAS, ROS1, and ALK mutation cases while CENPF is up-regulated in ROS1 and ALK mutation cases. EGFR may be involved in the progression of NSCLC by regulating various biological processes [36]. In the study, the expression of CENPF is down- regulated in EGFR mutation cases. Further study will continue to explore the potential role of miR-1-3p and CENPF in monitoring KRAS, ROS1, ALK and EGFR treatment effectiveness.

Although the regulation of miRNA-mRNA involved in LUAD was comprehensively analyzed and experimentally verified in this study, there are still some deficiencies in this study, such as lack of studies and insufficient sample size on the mechanism of DE-miRNAs and DE-mRNAs. More researches are needed to address these questions.

## CONCLUSIONS

In summary, we have constructed two miRNA-mRNA regulatory pairs that may be involved in the pathogenesis of LUAD. In the future, it is possible to help the treatment and prognosis of LUAD by targeting the established miRNA-mRNA regulatory pairs.

## Supplementary Material

Supplementary Figure 1

Supplementary Tables
