# Supplementary material for: Identify miRNA-mRNA regulation pairs to explore potential pathogenesis of lung adenocarcinoma
Source: Aging (Albany NY). 2022 Oct 19;14(20):8357–73. doi: 10.18632/aging.204341 (PMC9648793; doi:10.18632/aging.204341)
Supplement: Supplementary Figure 1 [file aging-14-204341-s001.pdf]

# SUPPLEMENTARY FIGURE

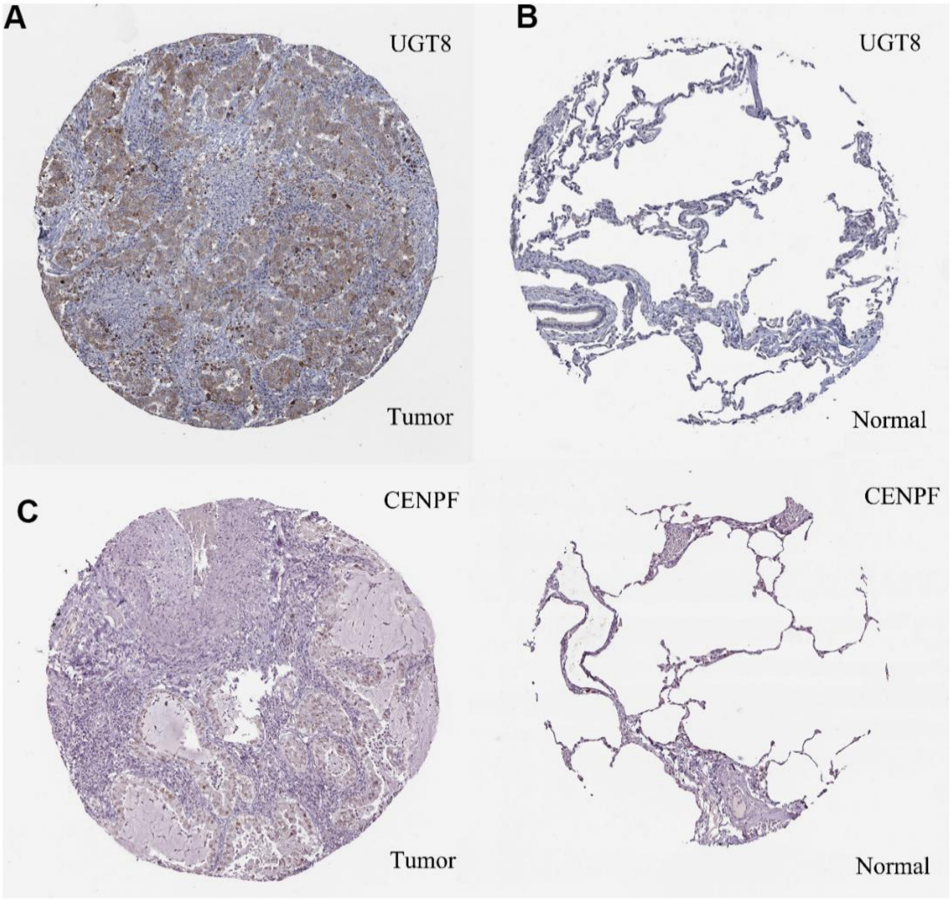


**Supplementary Figure 1. Immunohistochemistry images of UGT8 and CENPF in LUAD and normal lung tissue from HPA database.** (**A**) Medium immunostaining of UGT8 in LUAD cells (antibody HPA065785); (**B**) Immunostaining of UGT8 was not detected in normal lung tissue cells (antibody HPA065785); (**C**) Medium immunostaining of CENPF in LUAD cells (antibody CAB070134); (**D**) Immunostaining of CENPF was not detected in normal lung tissue cells (antibody CAB070134).
