# Supplementary material for: Identify miRNA-mRNA regulation pairs to explore potential pathogenesis of lung adenocarcinoma
Source: Aging (Albany NY). 2022 Oct 19;14(20):8357–73. doi: 10.18632/aging.204341 (PMC9648793; doi:10.18632/aging.204341)
Supplement: Supplementary Tables [file aging-14-204341-s002.pdf]

SUPPLEMENTARY TABLES

Supplementary Table 1. The sequences of primers for candidate miRNAs and targeted mRNAs.

	Name	Forward primer sequences (5'→3')	Reverse primer sequences (5'→3')
miRNA mature	hsa-miR-1-3p	TGGAATGTAAAGAAGTATGT	universal reverse primer
	hsa-miR-9-5p	TCTTTGGTTATCTAGCTGTAT	universal reverse primer
	hsa-miR-126-5p	CATTATTACTTTTGGTACG	universal reverse primer
	hsa-miR-135b-5p	TATGGCTTTTCATTCCTATGT	universal reverse primer
	hsa-miR-196a-5p	TAGGTAGTTTCATGTTGTT	universal reverse primer
mRNA	BMPR2	CACTCAGTCCACCTCATTCATTT	TTGTTTACGGTCTCCTGTCAAC
	STARD13	CGAGGAGACAGAAATGGGTCA	TCCACTGCTTTCGCTGTGAAT
	UGT8	AGAGACATCGCCCCATCTAAT	TCAAACAGTTCGATTGCTGTCA
	CENPF	ACCTTCACAACGTGTTAGACAG	CTGAGGCTCTCATATTCGGCA
	ITGB4	GCAGCTTCCAAATCACAGAGG	CCAGATCATCGGACATGGAGTT
Reference gene	RUN6B	CGATAAAATTGGAACGATACAGA	ATTTGGACCATTTCTCGATTTGT
	GAPDH	ACAACTTTGGTATCGTGGAAGG	GCCATCACGCCACAGTTTC

Supplementary Table 2. Immune cells differentiated between tumor tissue and normal tissue in TCGA-LUAD.

Cell type	Tumor	Normal	logFC	*P* Value
T cells regulatory (Tregs)	0.0317	0.0028	3.489	3.1655E-22
NK cells resting	0.0066	0.0382	-2.532	7.9002E-22
Monocytes	0.0164	0.0564	-1.784	2.0356E-21
Plasma cells	0.0926	0.0108	3.101	3.0347E-21
Eosinophils	0.0017	0.0118	-2.839	2.1925E-18
T cells follicular helper	0.0248	0.0041	2.586	7.0704E-16
Neutrophils	0.0084	0.0278	-1.732	1.0869E-12
Macrophages M1	0.0662	0.0326	1.023	1.0209E-09
Mast cells resting	0.0545	0.0831	-0.610	1.7838E-08
T cells CD4 memory resting	0.1567	0.2052	-0.389	1.9722E-06
Macrophages M2	0.1520	0.1926	-0.341	2.0297E-06
T cells CD4 memory activated	0.0229	0.0038	2.576	4.3641E-06
Dendritic cells resting	0.0544	0.0206	1.400	4.3387E-05
T cells CD4 naive	0.0000	0.0000	/	2.7512E-03
B cells memory	0.0086	0.0026	1.748	3.4089E-03
Dendritic cells activated	0.0222	0.0299	-0.430	3.6920E-03
Macrophages M0	0.1431	0.1695	-0.245	6.9230E-03
T cells gamma delta	0.0044	0.0005	3.038	7.8111E-03
B cells naive	0.0243	0.0158	0.616	1.0380E-02

FC, Fold change.
